# Ciclopirox activates PERK-dependent endoplasmic reticulum stress to drive cell death in colorectal cancer

**DOI:** 10.1038/s41419-020-02779-1

**Published:** 2020-07-27

**Authors:** Jianjun Qi, Ningning Zhou, Liyi Li, Shouyong Mo, Yidan Zhou, Yao Deng, Ting Chen, Changliang Shan, Qin Chen, Bin Lu

**Affiliations:** 1 Protein Quality Control and Diseases laboratory, School of Laboratory Medicine and Life Sciences, Wenzhou Medical University, Wenzhou, Zhejiang 325035 China; 2 Department of Intensive Care, The First Affiliated Hospital of Wenzhou Medical University, Wenzhou, Zhejiang 325000 China; 3 Department of Surgery, The Second Affiliated Hospital of Wenzhou Medical University, Wenzhou, Zhejiang 325000 China; 4 Department of Laboratory Medicine, The Fifth Affiliated Hospital of Wenzhou Medical University, Lishui, Zhejiang 32300 China; 5 State Key Laboratory of Medicinal Chemical Biology, College of Pharmacy and Tianjin Key Laboratory of Molecular Drug Research, Nankai University, Tianjin, 300350 China

**Keywords:** Colorectal cancer, Apoptosis, Energy metabolism

## Abstract

Ciclopirox (CPX) modulates multiple cellular pathways involved in the growth of a variety of tumor cell types. However, the effects of CPX on colorectal cancer (CRC) and the underlying mechanisms for its antitumor activity remain unclear. Herein, we report that CPX exhibited strong antitumorigenic properties in CRC by inducing cell cycle arrest, repressing cell migration, and invasion by affecting N-cadherin, Snail, E-cadherin, MMP-2, and MMP-9 expression, and disruption of cellular bioenergetics contributed to CPX-associated inhibition of cell growth, migration, and invasion. Interestingly, CPX-induced reactive oxygen species (ROS) production and impaired mitochondrial respiration, whereas the capacity of glycolysis was increased. CPX (20 mg/kg, intraperitoneally) substantially inhibited CRC xenograft growth in vivo. Mechanistic studies revealed that the antitumor activity of CPX relies on apoptosis induced by ROS-mediated endoplasmic reticulum (ER) stress in both 5-FU-sensitive and -resistant CRC cells. Our data reveal a novel mechanism for CPX through the disruption of cellular bioenergetics and activating protein kinase RNA-like endoplasmic reticulum kinase (PERK)-dependent ER stress to drive cell death and overcome drug resistance in CRC, indicating that CPX could potentially be a novel chemotherapeutic for the treatment of CRC.

## Introduction

Colorectal cancer (CRC) is the third most commonly diagnosed cancer and is the second cause of cancer-related death worldwide^1,2^. Chemotherapy combined with radiation therapy is the main treatment strategies used for advanced CRC. Primary chemotherapeutic regimens include 5-fluorouracil (5-FU), oxaliplatin and/or leucovorin, folinic acid and irinotecan (FOLFIRI), however, nearly all patients with metastatic lesions develop chemoresistance and the 5-year survival rate after surgical removal of CRC remains low^3^. Therefore, finding novel treatments for CRC remains a focus of anticancer research.

Ciclopirox (CPX; 6-Cyclohexyl-1-hydroxy-4-methyl-2(1H)-pyridinone) is a broad-spectrum hydroxypyridone-based synthetic topical antifungal with anti-inflammatory effects^4^. CPX was widely used as an antifungal agent for decades and has been used as CPX olamine, the olamine (2-aminoethanol) salt of CPX^5^. Interestingly, CPX has recently been identified as a potential anticancer agent for a variety of cancers^6–9^. CPX was found to inhibit rhabdomyosarcoma by inhibiting mTORC1 signaling through the activation of the AMPK pathway^10^. CPX also has an antileukemic and -neuroblastic effect by inhibiting β-Catenin and c-Myc signaling^11,12^. In addition, CPX could induce autophagy by reactive oxygen species (ROS)-mediated JNK activation^13^. Recently, CPX was found to trigger ROS-induced cell death and inhibit the cytoprotective autophagy in CRC cells through downregulating DJ-1 expression^14^. Moreover, CPX may direct interact with human high-mobility group A2 and thus inducing CRC cell cycle arrest and apoptosis^15^. As an iron chelator, CPX has also been found to inhibit leukemia and myeloma cells by disrupting iron metabolism^8^. In a phase I clinical trial, CPX showed efficacy and tolerability in the treatment of patients with relapsed or refractory hematologic malignancies^16^. Even though there is accumulating evidence regarding CPX’s antitumor activity, the data are still limited and the underlying molecular mechanisms remain largely unknown, which hinders the use of CPX in preclinical and clinical studies.

The underlying mechanisms of CPX-induced endoplasmic reticulum (ER) stress and the subsequent activation of protein disulfide isomerase (PDI) and ER oxidoreductase 1 (Ero1) in CRC cells has not been reported yet. A variety of stimuli can lead to the accumulation of misfolded or unfolded proteins in the ER, which leads to activation of the unfolded protein response (UPR) in an effort to relieve cell stress or initiate apoptosis^17–19^. The proteasome plays a critical role in the clearance of unfolded/misfolded proteins; emerging evidence suggests that targeting proteasomes is a promising treatment strategies for cancer due to its ability to selectively kill malignant cells^20^. Accumulation of misfolded/unfolded proteins in the ER lumen triggers the UPR, which halts protein translation, recruits ER chaperones, and promotes protein degradation to reduce unfolded proteins. The UPR sensor in the ER requires inositol-requiring enzyme 1α, protein kinase RNA-like endoplasmic reticulum kinase (PERK), and activated transcription factor 6 to monitor protein folding in the ER and globally repress protein synthesis until protein folding ability is recovered^21^. Upon activation, PERK directly phosphorylates and activates the ubiquitous eukaryotic translation initiation factor 2α (eIF2α), therefore, reducing the load of ER protein folding^22,23^. Phosphorylation of eIF2α facilitates translation of mRNA encoding the transcription factor (ATF4). ATF4 translocates to the nucleus and induces expression of ER chaperones, such as PDI, thereby increasing refolding of misfolded proteins^24^. PDI is an ER chaperone induced during ER stress and is responsible for the formation of disulfide bonds in proteins. Accumulation of misfolded or unfolded proteins disrupts ER homeostasis and causes ER stress^25^. PDI can be regenerated to its oxidized form in the ER by transferring electrons to reoxidizing proteins, such as Ero1^26^. The underlying mechanisms of CPX-induced ER stress and the subsequent activation of PDI and Ero1 in CRC cells has not been reported yet.

In the present study, we systematically investigated the role and underlying mechanisms of CPX suppression of CRC cell growth both in vitro and in vivo, to determine if CPX should be repurposed as an anticancer agent for CRC patients.

## Materials and methods

### Cell lines and cell culture

Human CRC cell lines HCT-8, HCT-8/5-FU, and DLD-1 were purchased from the Cell Bank of Shanghai Institute of Cell Biology (Shanghai, China). HCT-8 and DLD-1 cells were maintained in RPMI 1640 medium (Life Technologies, Grand Island, NY, USA) supplemented with 10% (v/v) fetal bovine serum (FBS, Life Technologies, Grand Island, NY, USA) and antibiotics (100 IU/mL penicillin and 100 μg/mL streptomycin) while HCT-8/5-FU cells were cultured in RPMI 1640 medium with 15 μM 5-FU. All cell lines were incubated at 37 °C with 5% CO_2_ in a humidified atmosphere unless otherwise noted. Cell lines were authenticated by short tandem repeats profiling before use and were also routinely tested for mycoplasma infection during this study.

### Reagents and antibodies

Cell Counting Kit-8 (CCK-8), crystal violet, and NAC (N-acetyl-L-cysteine) were purchased from Beyotime Biotechnology (Shanghai, China). Cell-cycle analysis kit was purchased from KeyGEN BioTECH (Jiangsu, China). The annexin V-fluorescein isothiocyanate (FITC)/propidium iodide (PI) apoptosis detection kit and PI/RNase staining buffer was purchased from BD Biosciences (San Jose, CA, USA). The bicinchoninic acid protein assay kit, Pierce ECL Western Blotting substrate, MitoSOX^TM^ Red mitochondrial Superoxide Indicator, 2-NBDG and TRIzol^TM^ Reagent, and Lipofectamine 3000 were purchased from Thermo Fisher Scientific (Waltham, MA, USA). Protease (Complete Mini) and phosphatase (PhosphoSTOP^TM^) inhibitor cocktail tablets were purchased from Roche Applied Science (Indianapolis, IN, USA). Matrigel and Transwell assays were purchased from Corning Incorporated (Corning, NY, USA). Seahorse XF96 V3 PS Cell Culture Microplates, Seahorse XFe96 FluxPak, Seahorse XF DMEM medium, Seahorse XF Calibrant Solution were purchased from Agilent Technologies Incorporated (Palo Alto, CA, USA). l-glutamine, glucose, sodium pyruvate, oligomycin, antimycin A, rotenone, carbonylcyanide-p-trifluoromethoxyphenylhydrazone (FCCP), 2-deoxyglucose, dimethyl sulfoxide (DMSO), and 5-FU were purchased from Sigma (St. Louis, MO, USA). CPX olamine was purchased from Dibai Biotechnology Co. (Shanghai, China). DNA extraction reagent was purchased from Solarbio Science & Technology Co., Ltd (Beijing, China). Enhanced chemiluminescence (ECL) kit was purchased from Perkin Elmer Life Science (Boston, MA, USA). The primary antibodies used in this study are provided in Table S1.

### Cell viability and proliferation assay

For the cell viability assay, cells were seeded in triplicates into a 96-well plate at a density of 5 × 10^3^ cells per well and cultured overnight. Cells were then treated with vehicle control (DMSO) or increasing concentrations of CPX (0, 5, 10, 20, 40, and 80 μM) for 48 h and cell viability was analyzed by CCK-8 assay according to manufacturer’s instructions. Briefly, CCK-8 reagent was added at a dilution of 1:10 to each well and incubated for 2–4 h. Optical density values were detected at a wavelength of 450 nm using a microplate reader (Varioskan^TM^ LUX Multi-Plate Reader, Thermo Fisher Scientific, Waltham, MA, USA). For the cell proliferation assay, 2 × 10^3^ cells were seeded into 96-well plates and cultured at overnight. The next day, cells were treated with different concentrations of CPX, as indicted above, or DMSO for 24, 48, and 72 h, and the relative cell number was measured by CCK-8 assay.

### Colony formation assay

A total of 800 cells per well were plated into six-well plates and incubated at 37 °C with 5% CO_2_. The medium was changed every 2 days until several cell clusters are visible under the microscope, and then the medium was replaced with fresh RPMI 1640 medium containing different concentrations of CPX as indicated or vehicle control (DMSO). The cells were cultured until the macroscopic clone was visible and then stained with a 0.5% crystal violet solution as previous described^27^. Visible colonies were counted and representative views were photographed.

### In vitro cell migration and invasion assays

For cell migration assay, HCT-8 (2 × 10^4^), HCT-8/5-FU (4 × 10^4^), and DLD-1 (6 × 10^4^) cells were seeded in the upper chamber of 24-well Transwell plates in serum-free RPMI 160 medium, respectively. The lower chamber contained 650 μl of RPMI 160 medium with 15% FBS. After cellular attachment, the medium in the upper chamber was replenished with serum-free medium containing increasing concentrations of CPX or vehicle control (DMSO). After 48 h, the cells that migrated to the lower chamber were fixed for 30 min in methanol and then stained with 0.5% crystal violet. Five randomly selected fields from each Transwell were photographed and analyzed using ImageJ Plus software.

For cell invasion assay, Matrigel was diluted 1:10 with precooled F-12 medium after thawed and liquefied on ice. Then 40 μl of Matrigel mixture was added to the upper chamber and incubated overnight. Next day, warm F-12 medium (50 μl) was added the upper chamber and incubated for 30 min to rehydrate the Matrigel layer. Subsequent procedures were the same as cell migration assay.

### Cell cycle and apoptosis assays by FACS

For cell-cycle analysis, HCT-8, HCT-8/5-FU, and DLD-1 cells were seeded in 6-cm dishes and cultured overnight, followed by treatment with vehicle control (DMSO) or a gradient concentration of CPX next day. After 24 h, cells were harvested and fixed in 70% ethanol and then stained with PI following RNase treatment. The stained cells were analyzed for cell-cycle distributions. For apoptosis assay, HCT-8, HCT-8/5-FU, and DLD-1 cells were incubated overnight, then treated with vehicle control (DMSO) or indicated concentration of CPX for 48 h. Cells were then collected and stained with Annexin V-FITC/PI for 20 min at room temperature.

Both cell-cycle distribution and cell apoptosis percentages were analyzed by flow cytometry on a BD Accuri^TM^ C6 plus flow cytometer (BD Biosciences, Franklin Lakes, NJ, USA).

### Mitochondrial and cellular reactive oxygen species (ROS) assays

To determine the effect of CPX on generation of ROS in CRC cells, cells (HCT-8, HCT-8/5-FU, and DLD-1 cells) were seeded in 6-cm cell culture dishes at a density of 4 × 10^5^ in RPMI 1640 medium and cultured overnight. Cells were then replaced with medium containing vehicle control (DMSO) or a gradient concentration of CPX. After 48 h of incubation, cells were harvested and the intracellular ROS were determined using DCFH-DA fluorescent probe according to the manufacturer’s protocol. Stained cells were analyzed using a flow cytometer (BD Accuri^TM^ C6 plus). Mitochondrial ROS were measured by staining cells with 5 μM MitoSOX^TM^ Red Mitochondrial Superoxide Indicator and stained cells were analyzed using a flow cytometer (BD Accuri^TM^ C6 plus).

### Western blotting analysis

Total proteins (15 μg per well) from lysed cells were separated by SDS-polyacrylamide gel electrophoresis and transferred onto nitrocellulose membranes. Membranes were incubated with specific primary antibodies overnight at 4 °C and HRP-conjugated secondary antibody for 1 h at room temperature. β-actin was employed as a protein loading control. Protein expression was visualized with ECL and exposed to X-ray film (Carestream Health, Xiamen, China). Protein expression levels in cells were quantified by ImageJ software.

### Mitochondrial respiration and glycolysis analysis

The Seahorse XF96 Extracellular Flux Analyzer (Agilent Technologies, Inc.) was used to measure the real-time oxygen consumption rate (OCR) and extracellular acidification rate (ECAR) of CRC cells according to the manufacturer’s instructions. Briefly, HCT-8, HCT-8/5-FU, and DLD-1 cells were seeded into 96-well cell plates, and incubated overnight. Meanwhile, the calibration plate was incubated at 37 °C, in a non-CO_2_ incubator overnight. Cells were pretreated with vehicle control (DMSO) or different concentration of CPX for 8 h, followed by replacing the medium with assay medium, and running the protocol until the calibration was completed, then OCR and ECAR were measured as previously described^28^.

### Glucose uptake assay

HCT-8 (4 × 10^5^), HCT-8/5-FU (5 × 10^5^), and DLD-1 (5 × 10^5^) cells were plated into 6-cm cell culture dishes and cultured overnight and then the medium, containing CPX or vehicle control (DMSO), was replenished. After 48 h, cells were harvested and glucose uptake was measured using 2-NBDG by flow cytometry.

### Mitochondrial DNA copy numbers detection

The cells were split using cracking mixture (ratio of DNA lysate and proteinase K is 100:1) at 55 °C overnight. Total DNA was extracted following the manufacturer’s instructions. qRT-PCR analysis was performed to measure the mitochondrial DNA (mtDNA) copy numbers using SYBR Green kit (Bio-Rad, Hercules, CA, USA) according to the manufacturer’s protocol on the CFX Connect^TM^ real-time system (Bio-Rad, Hercules, CA, USA). 18S ribosomal DNA was used as an internal control for all samples. The primer sequences for mtDNA (Cyt b) and 18S ribosomal DNA are listed in Table S2.

### In vivo subcutaneous xenograft models

Male nude mice were housed under specific pathogen-free conditions. HCT-8 (5 × 10^6^), HCT-8/5-FU (5 × 10^6^), and DLD-1 (5 × 10^6^) cells were subcutaneously injected into the left flank of 5 weeks old Balb/c nude mice (*n* = 12 per cell line), respectively. When the tumors reached ~100 mm^3^, mice were randomly divided into two groups of six [physiological saline (0.9% NaCl) group and CPX-treated group]. The mice then received an intraperitoneal injection of CPX (20 mg/kg), dissolved in 0.9% NaCl, or 0.9% NaCl alone (saline control), once a day for 12 days. Tumor volume was evaluated at indicated time points using the formula: volume = 1/2 × L × W^2^. Gross weight of each mouse was assessed every 2 days. After 12 days, the mice were sacrificed and photographed, then tumors were dissected, weighed, fixed, and embedded. All animal experiments were carried out in accordance with the Institutional Animal Care and Use Committee, University Laboratory Animal Research of Wenzhou Medical University.

### Immunohistochemistry

Immunohistochemistry (IHC) was performed as described previously^27^.

### Statistical analysis

All experiments in this study were performed in triplicate and repeated at least three times, unless otherwise indicated. Student’s *t* test was used to compare the mean between two groups, and the graphs were created by GraphPad Prism 7.0 Plus software (GraphPad Software Inc., San Diego, CA, USA). Data were expressed as mean ± SD, and *p* < 0.05 was considered statistically significant (*p < 0.05, **p < 0.01, ***p < 0.001; ns, no significant difference). Statistical analysis was carried out using SPSS software version 22.0 (SPSS Inc., Chicago, IL, USA).

## Results

### CPX inhibits CRC cell growth in vitro

**Fig. 1:**
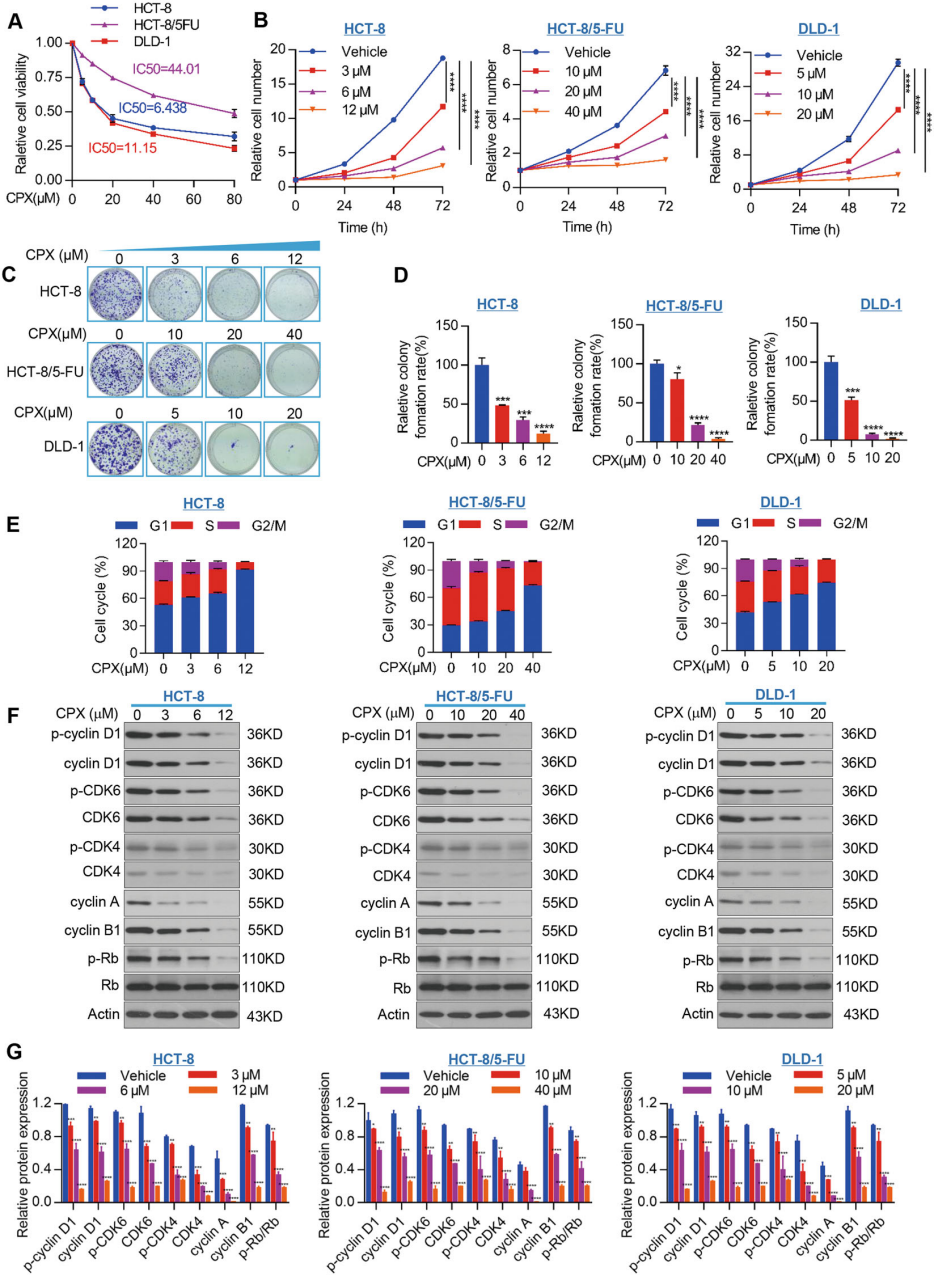
CPX inhibits CRC cell growth. **a** HCT-8, HCT-8/5-FU, and DLD-1 cells were plated in 96-well plates and treated with the indicated concentration of CPX or DMSO for 48 h. The CCK-8 kit was used to measure the relative cell viability. **b** CRC cell lines were plated in 96-well plates and treated with CPX with the indicated concentration or DMSO. Cell growth was assessed at 24, 48, and 72 h by CCK-8 assay. Colony-forming ability assay of HCT-8, HCT-8/5-FU, and DLD-1 cells treated with CPX or DMSO for 7 days. The cell colonies were stained with crystal violet solution (**c**) and the colony numbers were counted using ImageJ Plus software (**d**). **e** Cell-cycle analysis of cells treated with CPX with the indicated concentration or DMSO for 24 h. Cell-cycle distributions were analyzed by flow cytometry. **f** The western blotting analysis of the expression of cell cycle-related proteins in cells treated with indicated concentration of CPX or DMSO for 48 h. **g** Quantitative data of indicated cell cycle-related proteins in (**f**). All data are presented as the mean ± SD (*n* = 3, **p < 0.01; ***p < 0.001; ****p < 0.0001).

To evaluate the anticancer activity of CPX in CRC cells, we performed cellular proliferation and viability assays. Briefly, CRC cell lines (HCT-8, HCT-8/5-FU and DLD-1) were treated with CPX at concentrations of 5, 10, 20, 40, 80 μM or vehicle control (DMSO) for 48 h and cell viability was assessed using CCK-8 assays. In addition, we treated CRC cell lines with indicated concentration of CPX or vehicle control (DMSO) and relative cell numbers were measured at 24, 48, and 72 h later using CCK-8 assay. The results showed that CPX markedly suppressed CRC viability and proliferation in vitro (Fig. 1a, b). To further evaluate the antiproliferative activity of CPX, we performed a colony formation assay. As shown in Fig. 1c, d, CPX (HCT-8 cells: 0, 3, 6, and 12 μM; HCT-8/5-FU cells: 0, 10, 20, 40 μM; DLD-1: 0, 5, 10, 20 μM) treatment significantly reduced the colony-forming ability of CRC cells in a dose-dependent manner. Moreover, we found CPX treatment led to cell cycle arrest in G1 phase (Figs. 1e and S1).

To further investigate the mechanism of CPX’s anticancer activity in CRC, we examined the expression of cell cycle-related proteins in CPX-treated CRC cells. The results showed that CPX treatment significantly reduced the levels of cell cycle-related proteins. Cyclin A, cyclin D1, cyclin B1, CDK4, and CDK6 were significantly reduced in CRC cells treated with CPX for 48 h (Fig. 1f, g). In addition, the active form of CDKs including p-cyclin D1, p-CDK4, and p-CDK6 were also significantly downregulated in CRC cells following CPX treatment (Fig. 1f, g). As expected, the protein level of p-Rb/Rb was reduced remarkably (Fig. 1f, g). These results together indicate that CPX’s antitumorigenic activity in CRC cells is through arresting cell cycle.

### CPX inhibits tumor growth in vivo in a mouse xenograft model of CRC

**Fig. 2:**
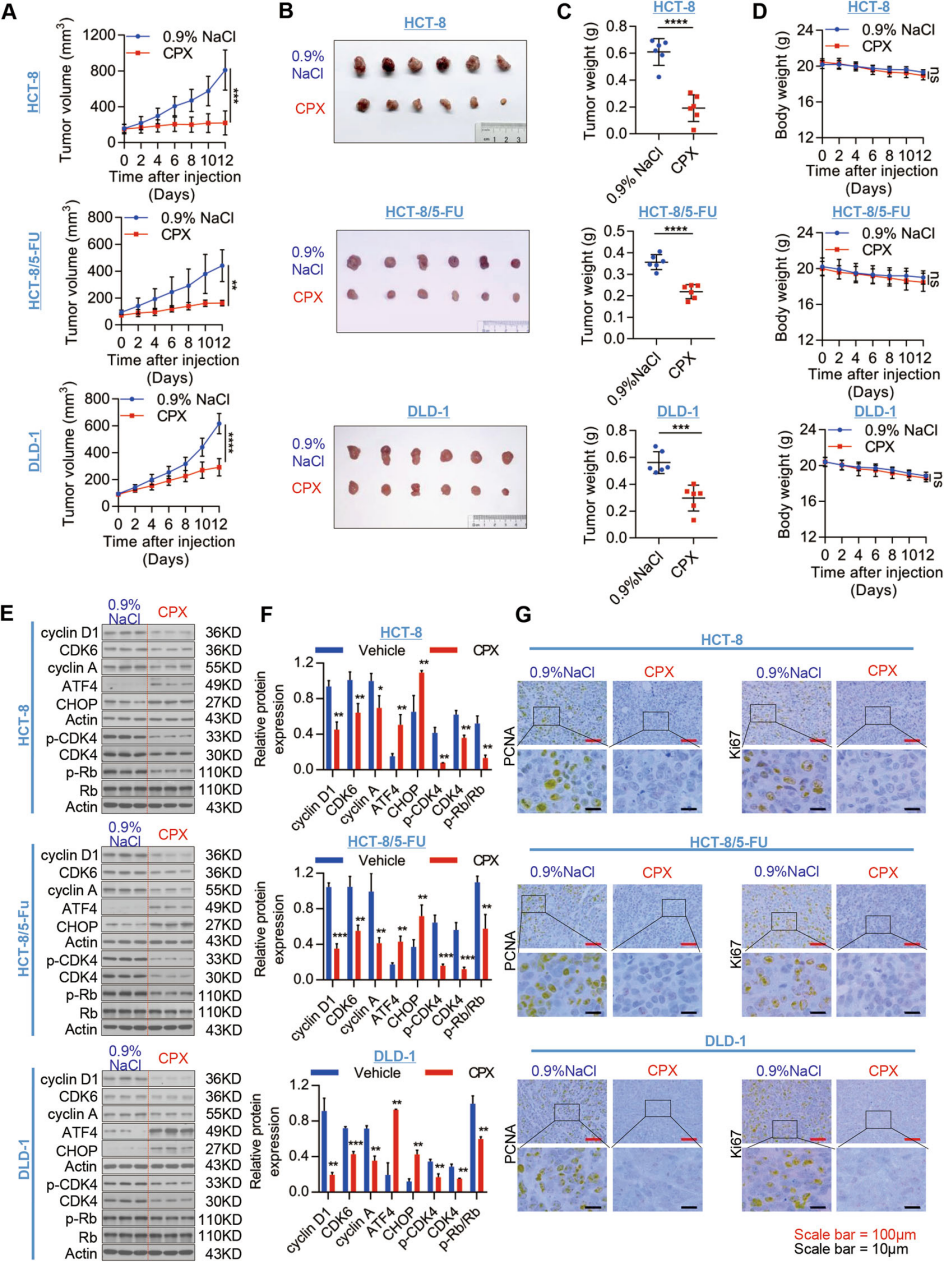
CPX inhibits tumor growth in vivo in a mouse xenograft model of CRC. **a**–**c** Two groups of tumor-bearing nude mice (*n* = 6, each group) were injected with either CPX (20 mg/kg) or physiological saline (0.9% NaCl), respectively. Tumor volumes from each group were evaluated at the indicated time points (**a**). Data are presented as the mean ± SD (*n* = 6, **p < 0.01; ***p < 0.001; ****p < 0.0001). After 12 days of consecutive injections, the mice were sacrificed. Representative images of dissected tumors are shown (**b**) and tumor weight was measured (**c**). Data are presented as the mean ± SD (*n* = 6, ***p < 0.001; ****p < 0.0001). Changes in mean body weight after CPX or 0.9% NaCl treatment for the HCT-8, HCT-8/5-FU, and DLD-1 xenograft model (**d**). Data are presented as the mean ± SD (*n* = 6, ns not significant for indicated comparison). CDK4, p-CDK4, CDK6, cyclin A, cyclin D1, Rb, p-Rb, ATF4, and CHOP protein expression in tumor tissue lysate was examined by western blot analysis (**e**) and quantified using ImageJ Plus software (**f**). Data are presented as the mean ± SD (*n* = 3, *p < 0.05; **p < 0.01; ***p < 0.001). **g** IHC staining of PCNA and Ki-67 in tumor sections of mouse xenograft model of CRC cells treated with saline or CPX.

To further investigate the antitumor activity of CPX, a mouse xenograft model of CRC was employed to evaluate the activity of CPX in vivo. HCT-8, HCT-8/5-FU, and DLD-1 cells were injected subcutaneously into the left flank of 5-week-old mice (*n* = 12). When the tumor volume reached ~100 mm^3^, the mice were randomly divided into two groups (saline and CPX) with six mice per group. The CPX-treated groups received an intraperitoneal injection of 20 mg/kg CPX once per day for 12 consecutive days. At the same time, the saline group received the same volume of 0.9% NaCl (saline control). As shown in Fig. 2a, CPX significantly inhibited CRC cell growth when compared to control group. Consistent with these results, tumor weight was also reduced in the CPX-treated group (Fig. 2b, c). Treatment with CPX was well tolerated without notable body weight loss (Fig. 2d). We analyzed CDK4, CDK6, cyclin A, cyclin D1, ATF4, and CHOP protein levels in excised tumor sections from saline- and CPX-treated groups. CPX treatment was found to correlate with a dramatic downregulation of cyclin D1, CDK4, CDK6, and cyclin A expression with a concomitant increase in ATF4 and CHOP expression (Fig. 2e, f). Moreover, levels of p-CDK4 and p-Rb/Rb were both significantly downregulated (Fig. 2e, f). Consistent with its effects in vitro, CPX increased the tumor proliferative index (Fig. 2g) compared to 0.9% NaCl treatment as determined by IHC for PCNA and Ki-67, respectively. In conclusion, these results indicate that CPX inhibits CRC cell growth in vivo.

### CPX suppresses the migration and invasion of CRC cells

**Fig. 3:**
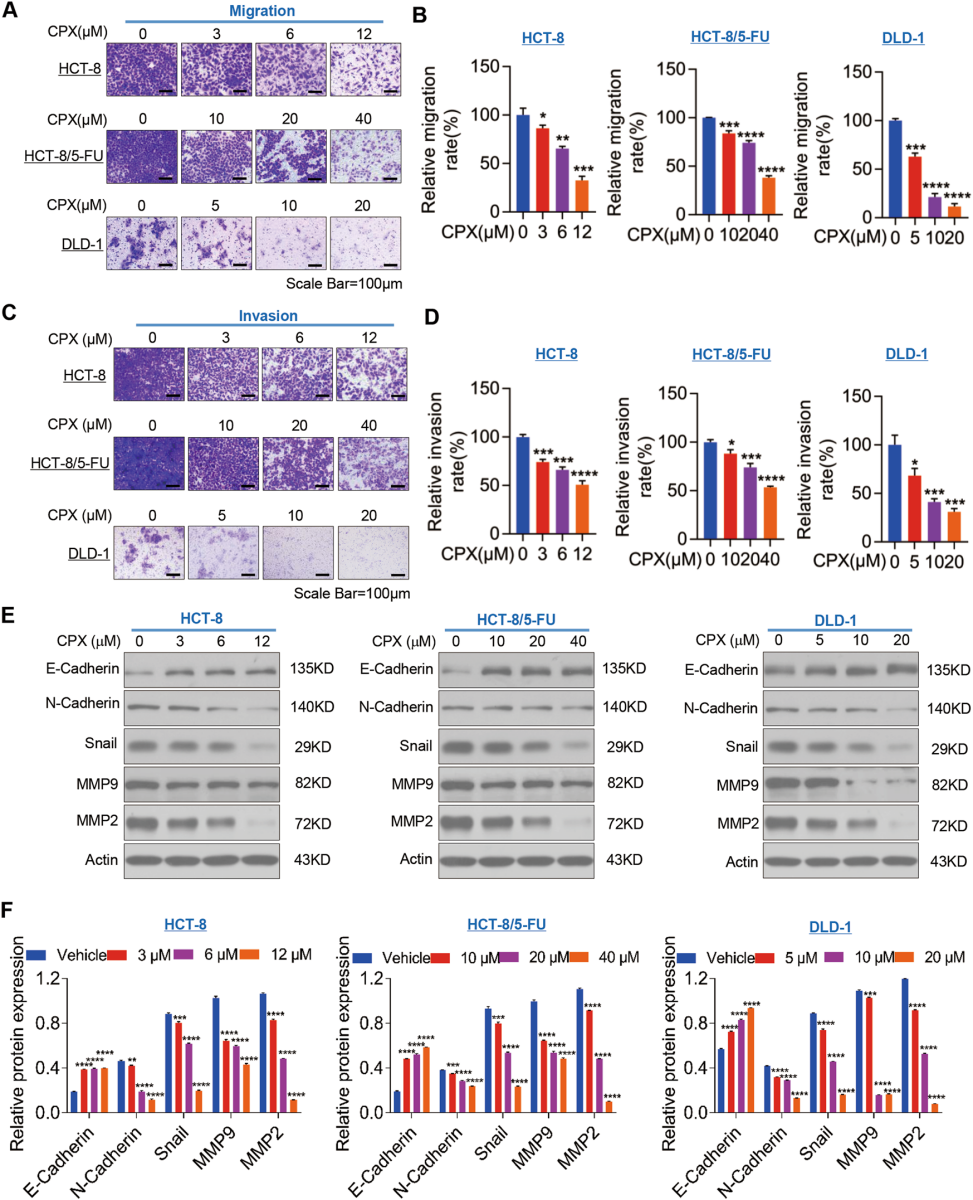
CPX suppresses the migration and invasion of CRC cells. **a**, **b** The Transwell cell migration assay of HCT-8, HCT-8/5-FU, and DLD-1 cells treated with vehicle or CPX at indicated concentration for 48 h. The migrated cells were visualized by crystal violet staining and observed with light microscope, and the representative images were shown (magnification, ×100) (**a**). The cell migration rate was quantified by ImageJ Plus (**b**). **c**, **d** The Transwell cell invasion assay of CRC cells treated with vehicle or CPX at indicated concentration. Invading cells were visualized with crystal violet staining, observed with a light microscope, and representative images are shown (magnification, ×100) (**c**). Cell invasion rate was quantified by ImageJ Plus (**d**). HCT-8, HCT-8/5-FU, and DLD-1 cells were treated with vehicle or CPX at indicated concentration for 48 h, and cells were then collected and subjected to western blotting analysis with indicated antibodies for cell migration and invasion related proteins (**e**) and the relative protein expression was quantitated by ImageJ Plus (**f**). Data are presented as the mean ± SD (*n* = 3, *p < 0.05; **p < 0.01; ***p < 0.001; ****p < 0.0001 when compared with vehicle control).

Metastasis remains the principal risk factor that contributes to a high mortality rate in CRC patients, therefore, we investigated the potential impacts of CPX on CRC cell migration and invasion. Our results using Transwell assays showed that CPX treatment significantly suppressed HCT-8, HCT-8/5-FU, and DLD-1 cell migration and invasion in a dose-dependent manner in vitro (Fig. 3a–d). Epithelial–mesenchymal transition (EMT) has been shown to play a crucial role in promoting metastasis and invasion of cancer cells, which led us to examine the expression of EMT markers, including E-cadherin, N-cadherin, and Snail. Moreover, the protein levels of matrix metalloproteinases (MMP-2 and -9) were also examined. Immunoblotting analysis showed that the protein expression of epithelial marker E-cadherin was increased, while the expression of mesenchymal markers N-cadherin and Snail were markedly reduced in CPX-treated CRC cells (Fig. 3e, f) and MMP-2 and MMP-9 expression levels were also decreased in a dose-dependent manner (Fig. 3e, f). Together, these results suggest that CPX suppresses the migration and invasion potential of CRC cells by modulating EMT.

### CPX impairs mitochondrial respiration and induces ROS production in CRC cells

**Fig. 4:**
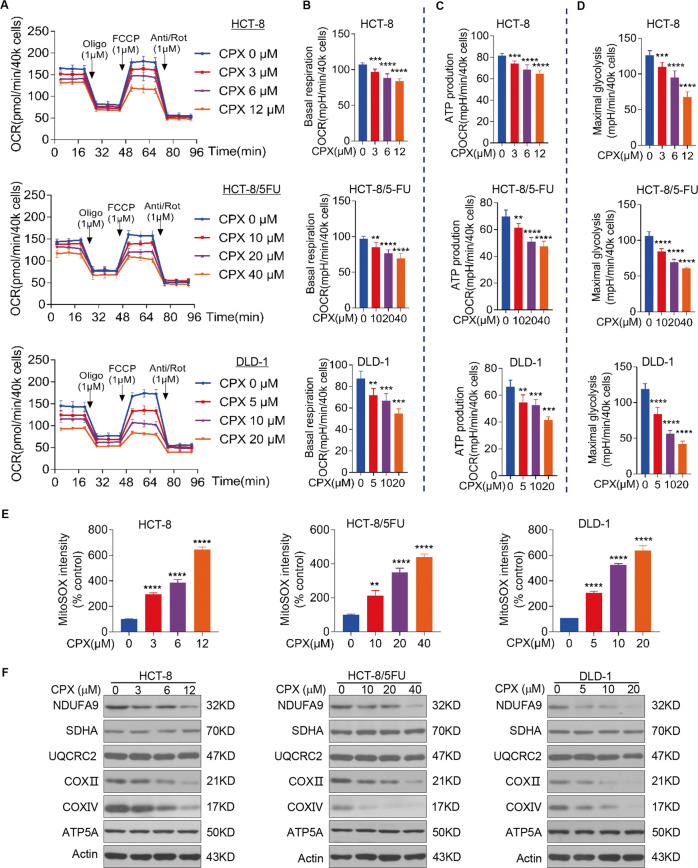
CPX impairs mitochondrial respiration and induces ROS production in CRC cells. **a** Overall OCR alteration of vehicle- or CPX-treated HCT-8, HCT-8/5-FU, and DLD-1 cells were analyzed by Seahorse XF96 bioenergetics analyzer. Basal respiration (**b**), ATP production associated OCR rate (**c**), and maximal respiration (**d**) of cell lines treated with vehicle or CPX at indicated concentration. **e** HCT-8, HCT-8/5-FU, and DLD-1 cells were treated with vehicle or CPX at indicated concentration and mitochondrial ROS (mitoROS) level was measured by flow cytometry. **f** CRC cell lines were treated with vehicle or CPX at indicated concentration for 48 h and cell samples were collected and followed by western blotting analysis with indicated antibodies. Data were presented as the mean ± SD (*n* = 3, **p < 0.01; ***p < 0.001; ****p < 0.0001 when compared with vehicle control). Oligo oligomycin (ATP synthase inhibitor), FCCP carbonylcyanide-p-trifluoromethoxyphenylhydrazone (mitochondrial OXPHOS uncoupler), Anti antimycin A (mitochondrial electron transport chain inhibitor), Rot rotenone (mitochondrial electron transport chain inhibitor).

Most of the cellular energy (ATP) is produced by mitochondrial oxidative phosphorylation (OXPHOS). Thus, targeting mitochondrial OXPHOS is considered as a promising therapeutic strategy for cancer. We next used the Seahorse XF96 flux analyzer to measure the OCR, a measurement of mitochondrial OXPHOS. Our results showed that the OCR was dramatically decreased after CRC cell lines were treated with increasing concentrations of CPX (Fig. 4a), suggesting that CPX treatment led to inhibition of mitochondrial respiration. We further assessed other parameters of mitochondrial function by analyzing OCR data at different time points and we found that CPX markedly decreased the basal respiration, maximal respiration, and ATP production in CRC cells (Fig. 4b–d). To delineate the underlying mechanisms, we first examined the ROS production in CRC cells upon CPX treatment. We found that CPX induced both mitochondrial and cellular ROS were significantly increased (Figs. 4e and S2D). Moreover, we observed that CPX treatment resulted in mtDNA reduced in a dose-dependent manner (Fig. S2B). We also observed that CPX treatment caused a decrease in the protein expression of a variety of mitochondrial respiratory chain enzyme subunits, including NDUFA9, COX II, and COX IV (Fig. 4f). Whereas the protein level of SDHA and ATP5A remained unchanged and a slight decrease in UQCRC2 expression was observed (Fig. 4f).

### CPX remarkably promotes aerobic glycolysis of CRC cells

**Fig. 5:**
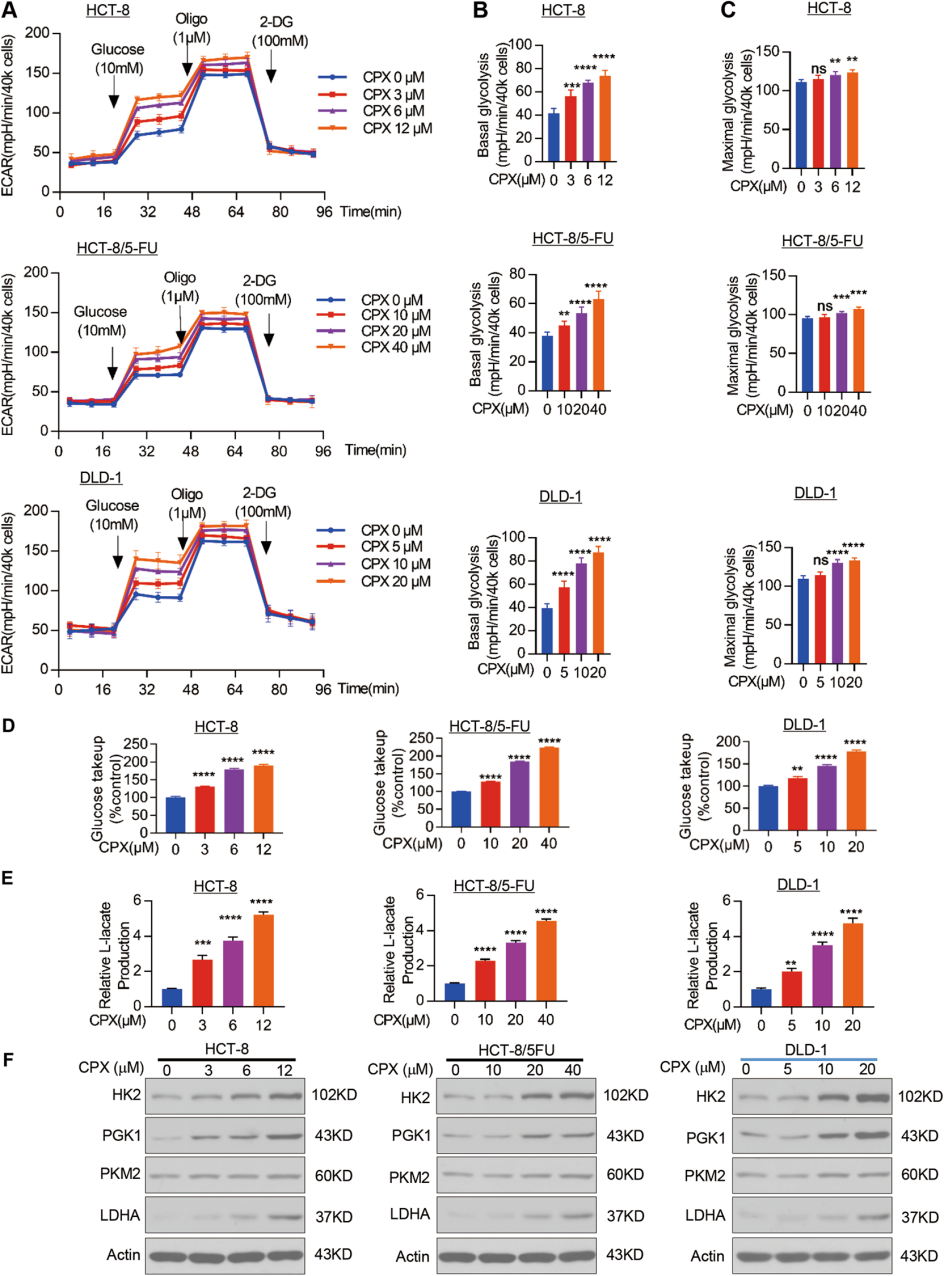
CPX promotes aerobic glycolysis in CRC cells. **a** Overall ECAR curves of HCT-8, HCT-8/5-FU, and DLD-1 cells treated with vehicle or CPX at indicated concentration. Basal glycolytic rate (**b**), maximal glycolytic rate (**c**), and spare glycolytic capacity (**d**), of HCT-8, HCT-8/5-FU, and DLD-1 cells treated with vehicle or CPX at indicated concentration. The relative glucose uptake (**e**) and lactate production (**f**) in HCT-8, HCT-8/5-FU, and DLD-1 cells treated with vehicle or CPX at indicated concentration. **g** Cells were treated with vehicle or CPX, and then collected and subjected to western blotting analysis with indicated antibodies. Data were presented as the mean ± SD (*n* = 3, **p < 0.01; ***p < 0.001; ****p < 0.0001 when compared with vehicle control; ns not significant for indicated comparison).

To explore the effects of CPX on glycolysis of CRC cells, we analyzed the ECAR of HCT-8, HCT-8/5-FU, and DLD-1 cells treated with CPX for 8 h. Our results showed that CPX significantly promoted the overall aerobic glycolytic rate in CRC cell lines (Fig. 5a). We further analyzed the indices that represent alteration of glycolysis and found that both the basal glycolysis and maximal glycolytic rate were remarkably increased (Fig. 5b, c). Consistent with the results that CPX promotes ECAR in CRC cells, we observed that both the uptake of glucose and lactate production were also increased in CPX-treated CRC cells in a dose-dependent manner (Fig. 5d, e). To further understand how CPX promotes the glycolysis in CRC cells, we analyzed the expression of several key enzymes in the glycolytic pathway including HK2, PGK1, and LDHA. The results showed that the protein levels of these key enzymes were all increased with CPX treatment compared to vehicle-treated CRC cells (Fig. 5f). These results revealed that CPX could promote aerobic glycolysis of CRC cells.

### CPX activates PERK-dependent ER stress

**Fig. 6:**
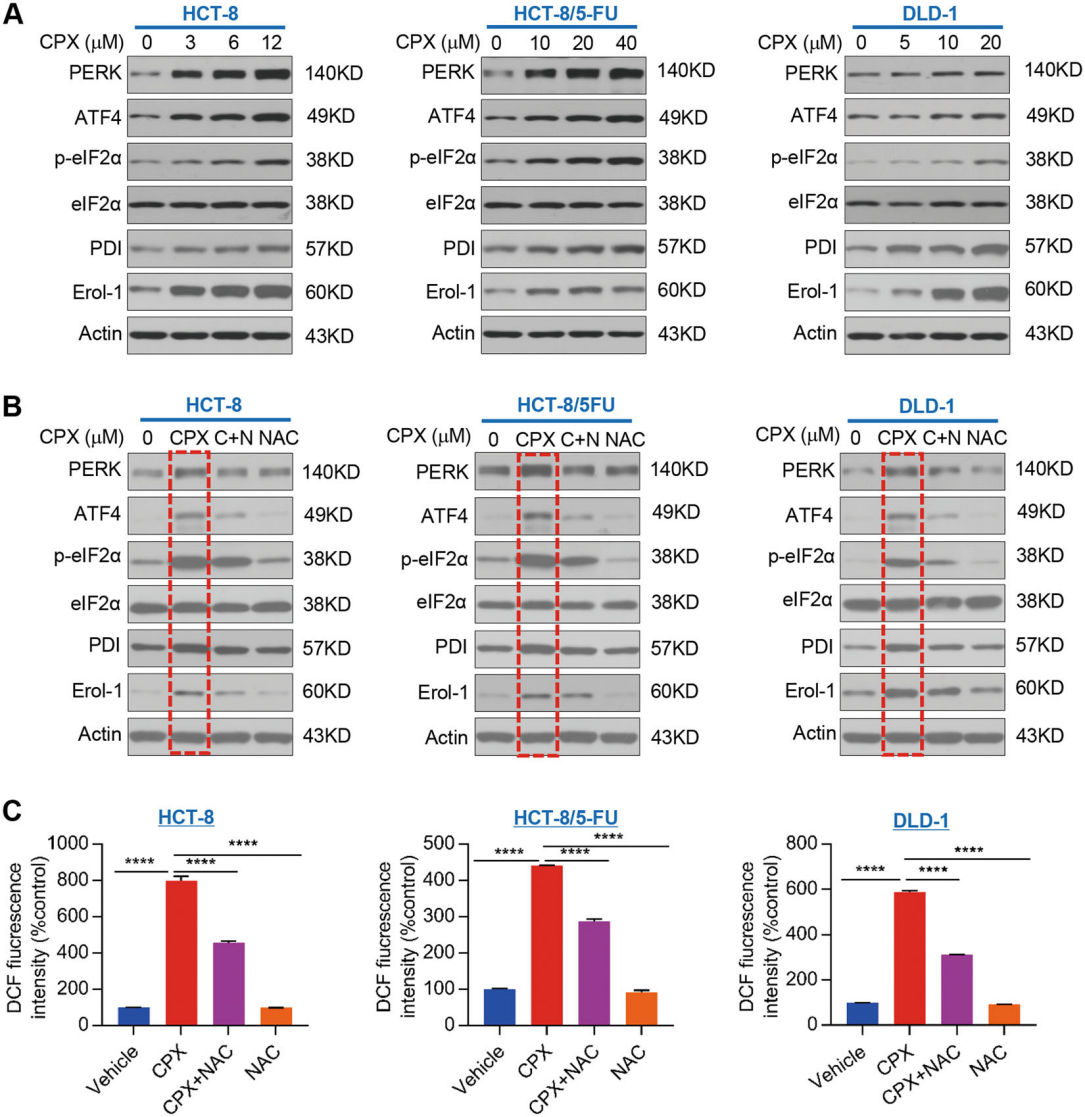
CPX activates PERK-dependent ER stress. **a** Expression levels of ER stress and UPR^ER^ markers were analyzed by western blotting in HCT-8, HCT-8/5-FU, and DLD-1 cells treated with vehicle or CPX. **b** HCT-8, HCT-8/5-FU, and DLD-1 cells were treated with vehicle, CPX (12, 40, 20 μM) or CPX combined with the ROS scavenger NAC (5 mM) or NAC alone (5 mM) for 48 h. The whole cell extracts were analyzed by western blotting using the indicated antibodies for ER stress and UPR^ER^ marker. **c** CRC cell lines were treated with vehicle or CPX, CPX combined with the ROS scavenger NAC (5 mM) or NAC alone (5 mM) for 48 h. The total cellular ROS level was measured by flow cytometry. Data were presented as the mean ± SD (*n* = 3, ****p < 0.0001).

To further explore the underlying mechanism by which CPX inhibits CRC cell growth, we next determined the effects of CPX on ER stress in CRC cells. As shown in Fig. 6a, CPX treatment significantly increased PERK expression compared to vehicle control in a dose-dependent manner. In addition, CPX treatment activated the phosphorylation of eIF2α, p-eIF2α, expression was significantly increased, while eIF2α protein level remains unchanged (Fig. 6a). CPX treatment also led to significant increases ATF4, PDI, and Ero1-Lα protein expression (Fig. 6a). However, pretreatment of CRC cells with ROS scavenger NAC rescued the effects of CPX on the expression of PERK, ATF4, PDI, Ero1-Lα, as well as activation of eIF2α (Fig. 6b). These data indicate that CPX treatment activates PERK-dependent ER stress in CRC cells and that this activation of ER stress by CPX is ROS-dependent. Pretreatment with the antioxidant NAC significantly attenuated the effect of CPX in promoting ROS production in CRC cells (Fig. 6c).

### CPX activates ER stress-induced apoptosis

**Fig. 7:**
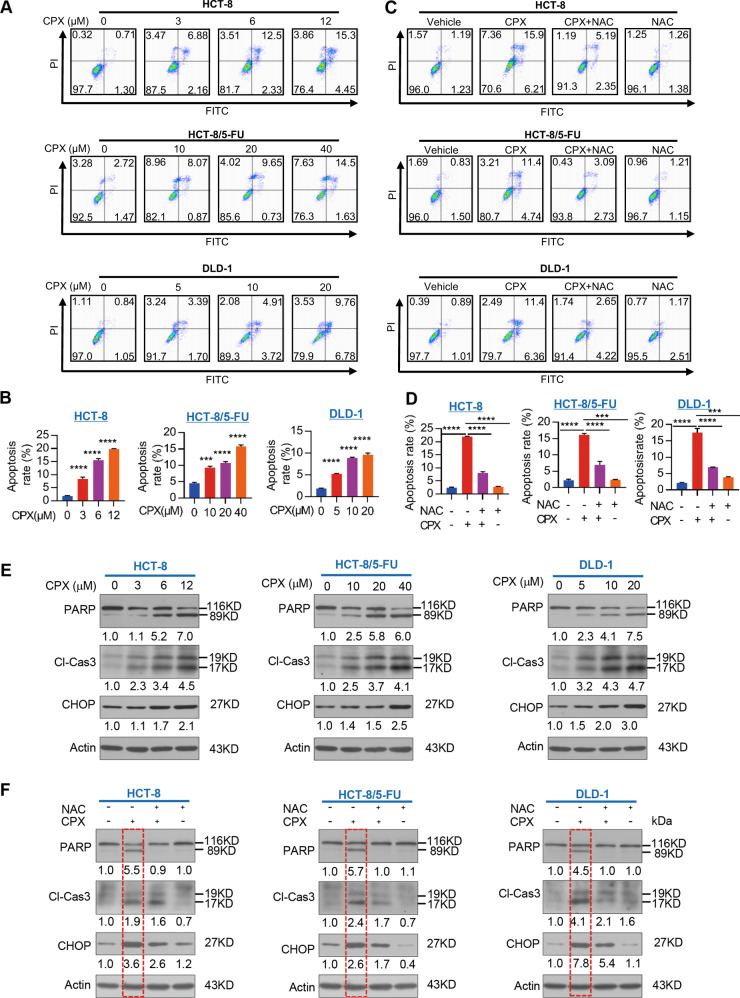
CPX activates ER Stress-induced apoptosis. **a**, **b** HCT-8, HCT-8/5-FU, and DLD-1 cells were treated with vehicle or CPX at indicated concentration for 48 h, and apoptosis was determined by flow cytometry. Representative images of apoptosis (**a**) and the quantification of apoptosis rate form three independent experiments (**b**). Data were presented as the mean ± SD (*n* = 3, ***p < 0.001; ****p < 0.0001). **c**, **d** CRC cell lines were treated with vehicle or CPX, or CPX combined with the ROS scavenger NAC (5 mM) or NAC alone (5 mM) for 48 h, and apoptosis was determined by flow cytometry. The representative images of apoptosis (**c**) and the quantification of apoptosis rate form three independent experiments (**d**). Data were presented as the mean ± SD (*n* = 3, ***p < 0.001; ****p < 0.0001). **e** Expression levels of PARP, caspase-3 cleavage as well CHOP were analyzed by western blotting in cells treated with vehicle or CPX at indicated concentration for 48 h. **f** Expression levels of PARP, caspase-3 cleavage, and CHOP were analyzed by western blotting in CRC cell lines treated with vehicle or CPX, or CPX combined with the ROS scavenger NAC (5 mM) or NAC alone (5 mM) for 48 h.

Since we observed that CPX treatment activates PERK-dependent ER stress in CRC cells, we speculated that CPX might activate ER stress-induced apoptosis in CRC cells. We next determined the effects of CPX in apoptosis of CRC cells by flow cytometry. The results showed that CPX treatment induced apoptosis in CRC cells (Fig. 7a, b) and that pretreatment of CRC cells with NAC significantly inhibited this effect (Fig. 7c, d). Moreover, we observed that CPX treatment resulted in PARP and caspase-3 cleavage as well as promoting CHOP expression in a dose-dependent manner (Fig. 7e), but pretreatment of with ROS scavenger NAC rescued CRC cells from this CPX-induced apoptosis and reversed the upregulation of CHOP (Fig. 7f). These results indicate that CPX inhibits CRC cell growth at least partially by inducing apoptosis through activating PERK-dependent ER stress and UPR^ER^.

## Discussion

Inducing cancer cell death by promoting apoptosis has been proposed as an effective therapeutic approach for cancer therapy^29^. The ER is an important organelle in eukaryotic cells and plays a critical role in the protein quality control to ensure that correctly folded proteins reach their destination^28,30^. Under certain circumstances, unfolded and/or misfolded proteins can accumulate within ER due to the limitation of the degradation capabilities of proteasomes, which leads to the activation of ER stress and UPR^ER^ to promote cell survival^31,32^. However, under conditions of severe and sustained ER stress, when the UPR^ER^ is unable to restore homeostasis of ER protein, the upregulation of CHOP expression results in apoptosis^33,34^. Thus, ER stress and UPR^ER^ signaling can be viewed as a double-edged sword; supporting cancer cell survival in an adverse environment, while promoting cancer cell death under severe and prolonged stress conditions^35,36^. ATF4 is a principle member of ATF/cyclic adenosine 3,5-monophosphate response element-binding family members and is regulated via the PERK pathway of eIF2α phosphorylation, which may facilitate ER protein homeostasis to support cellular survival or initiate apoptosis by upregulating the expression of proapoptotic BH3-only proteins and suppressing Bcl-2^21,32,37,38^.

CPX, an off-patent fungicide widely used for treatment of superficial mycoses, has also been found possessing anticancer effect on various types of cancer by inhibiting cell growth, inducing cell death as well as superessing angiogenesis^8,9,39^. However, the molecular mechanisms underlying the anticancer effect of CPX are still not fully understood. In this study, for the first time, we evaluated the role of CPX in the regulation of ER stress and UPR^ER^ signaling of CRC cells. Our results showed that CPX directly induces both mitochondrial and cellular ROS production, which leads to the activation of ER stress and UPR^ER^, which leads to activation of the PERK-eIF2α-ATF4 pathway, promoting ER stress-associated cell death in both chemoresistant (HCT-8/5-FU) and chemosensitive (HCT-8 and DLD-1) CRC cells. Interestingly, we found that the CPX-induced apoptosis could be rescued by the ROS scavenger NAC. Given that CPX-induced apoptosis was abolished by ROS scavenger, we speculated that ROS may be involved in the anticancer activity of CPX.

It is well known that enhancement of the glycolytic activity and decreased OXPHOS capacity is a phenomenon known as “Warburg effect,” which is considered as a critical hallmark of cancer^40–42^. However, recent studies give a better view of the changes in mitochondrial OXPHOS in tumorigenesis^43,44^. In a wide variety of cancer cell types, the mitochondria are able to efficiently synthesize ATP, thus choosing between glycolysis and OXPHOS is the tumor cell’s dilemma^45^. It has been reported that HeLa cells prefer to use OXPHOS to produce ATP for supporting growth in high glucose medium^46^ and mitochondrial OXPHOS generate almost 80% of the cellular ATP in HeLa cells growth in vitro^47^. These previous studies are in accordance with our findings, CPX significantly reduced the mitochondrial OXPHOS, leading to cellular bioenergetics catastrophe and apoptotic cell death in CRC cells. We observed that CPX impairs mitochondrial OXPHOS through enhancing mitochondrial ROS (superoxide) production. Consistent with the disruption of mitochondrial OXPHOS by CPX treatment, we observed that CPX treatment significantly suppressed the protein expression of several mitochondrial respiratory chain complex subunits including NDUFA9, COX II, and COX IV. Interestingly, we further demonstrated that CPX upregulates several key enzymes in the glycolytic pathway and thus promoting the glycolysis of CRC cells. Collectively, our results suggest that CRC cells mainly rely on OXPHOS rather than glycolysis, and mitochondrial respiration is a potential therapeutic target for CPX. Although further studies are required to fully understand how CPX inhibits mitochondrial OXPHOS and simultaneously promotes glycolysis in CRC cells, our data indicate this is a viable target for novel chemotherapeutic development.

CPX treatment leads to CRC cell cycle arrest at G1 phase through decreasing the expression of Cyclin A, Cyclin B1, Cyclin D1, CDK4, CDK6, as well as inhibiting the activation of CDKs and reducing the level of p-Rb/Rb, thereby suppressing CRC cell proliferation. Consistent with our in vitro data, CPX treatment dramatically decreased the protein expression of both PCNA and Ki-67, which are cell proliferation markers^48^, in the tumor sections of xenograft model of CRC compared with vehicle treatment (control group). EMT is essential for tumors acquiring aggressive features, such as invasiveness and metastatic ability^49^. MMPs also play critical roles in EMT to drive breast cancer progression^50^. Accordingly, CPX increases the EMT-related proteins such as E-cadherin expression, while reducing the expression of N-cadherin, Snail, MMP-2 and MMP-9, which suppressed cell migration and invasion ability of CRC cells.

**Fig. 8:**
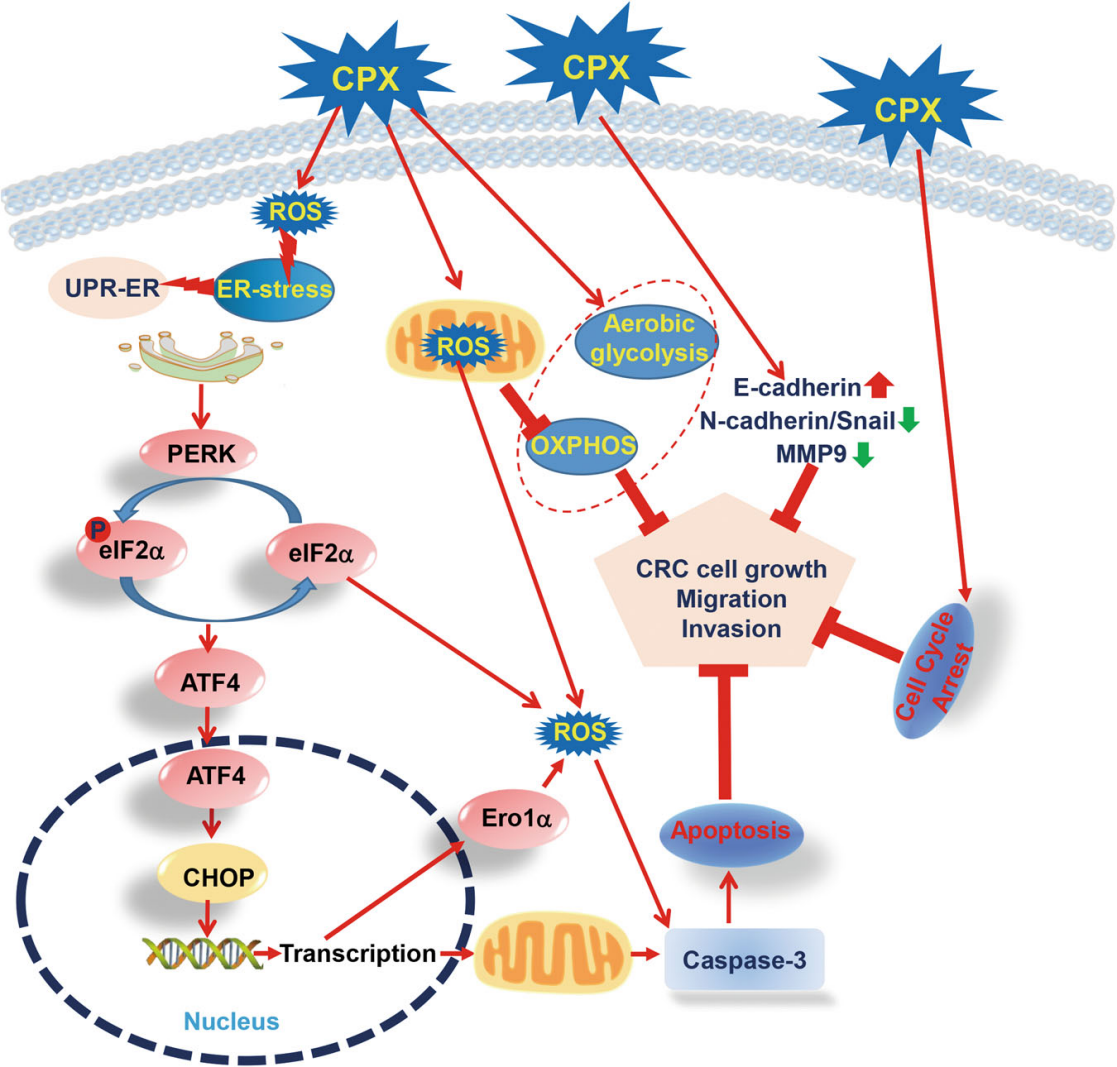
The proposed mechanistic model of CPX suppressing CRC cell growth, migration, and invasion. CPX exhibited antitumorigenic properties in CRC by inducing cell cycle arrest, inhibiting cell migration, and invasion by disrupting the expression of N-cadherin, Snail, E-cadherin, MMP-2, and MMP-9. CPX also induced ROS production and impaired mitochondrial OXPHOS, whereas the capacity of glycolysis was increased. Mechanistic studies revealed that the antitumor activity of CPX relies on apoptosis induced by ROS-mediated ER stress in CRC cells.

Throughout this study, we proposed a mechanistic model of CPX inhibitory activity in CRC cell proliferation, migration, and invasion (Fig. 8). CPX facilitates the activation of PERK-dependent ER stress and UPR^ER^ signaling to drive cell death in CRC. Taken together, our findings reveal a novel mechanism of the antineoplastic activity of CPX in CRC cells, which further supports the potential of CPX as an anticancer agent for CRC therapy. This promising approach may provide significant benefit to patients with other solid tumors.

## Supplementary information

Supplementary Figure Legends

Figure S1

Figure S2

Supplementary Table 1

Supplementary information
