# Correction: Ciclopirox activates PERK-dependent endoplasmic reticulum stress to drive cell death in colorectal cancer

**DOI:** 10.1038/s41419-020-02868-1

**Published:** 2020-09-03

**Authors:** Jianjun Qi, Ningning Zhou, Liyi Li, Shouyong Mo, Yidan Zhou, Yao Deng, Ting Chen, Changliang Shan, Qin Chen, Bin Lu

**Affiliations:** 1 Protein Quality Control and Diseases laboratory, School of Laboratory Medicine and Life Sciences, Wenzhou Medical University, Wenzhou, Zhejiang 325035 China; 2 Department of Intensive Care, The First Affiliated Hospital of Wenzhou Medical University, Wenzhou, Zhejiang 325000 China; 3 Department of Surgery, The Second Affiliated Hospital of Wenzhou Medical University, Wenzhou, Zhejiang 325000 China; 4 Department of Laboratory Medicine, The Fifth Affiliated Hospital of Wenzhou Medical University, Lishui, Zhejiang 32300 China; 5 State Key Laboratory of Medicinal Chemical Biology, College of Pharmacy and Tianjin Key Laboratory of Molecular Drug Research, Nankai University, Tianjin, 300350 China

Correction to: *Cell Death & Disease*

10.1038/s41419-020-02779-1 published online 27 July 2020

The original version of this Article omitted the statement “These authors contributed equally: Jianjun Qi and Ningning Zhou”. This has now been corrected in both the PDF and HTML versions of the Article.

