# Supplementary material for: Ciclopirox activates PERK-dependent endoplasmic reticulum stress to drive cell death in colorectal cancer
Source: Cell Death Dis. 2020 Jul 27;11(7):582. doi: 10.1038/s41419-020-02779-1 (PMC7385140; doi:10.1038/s41419-020-02779-1)
Supplement: Supplementary file 1 — Supplementary Figure Legends [file 41419_2020_2779_MOESM1_ESM.docx]

**Supplementary Figure Legends**

**Supplementary Figure 1. CPX caused cell cycle arrest in CRC cells.** HCT-8, HCT-8/5FU and DLD-1 cells were treated with vehicle control (DMSO) or indicated concentration of CPX for 48 h. Cell cycle distributions were analyzed by flow cytometry.

**Supplementary Figure 2. CPX induced cellular ROS generation and caused a reduction in mtDNA copy numbers.** a, b HCT-8, HCT-8/5FU and DLD-1 cells were treated with vehicle control (DMSO) or the indicated concentration of CPX for 48 h. Cellular ROS was analyzed by flow cytometry (a). mtDNA copy numbers were measured by qRT-PCR (b). Data are presented as the mean ± SD (n = 3, ***p* <0.01, ****p* < 0.001, *****p* < 0.0001).
