# Supplementary figures and images for: Ciclopirox activates PERK-dependent endoplasmic reticulum stress to drive cell death in colorectal cancer
Source: Cell Death Dis. 2020 Jul 27;11(7):582. doi: 10.1038/s41419-020-02779-1 (PMC7385140; doi:10.1038/s41419-020-02779-1)

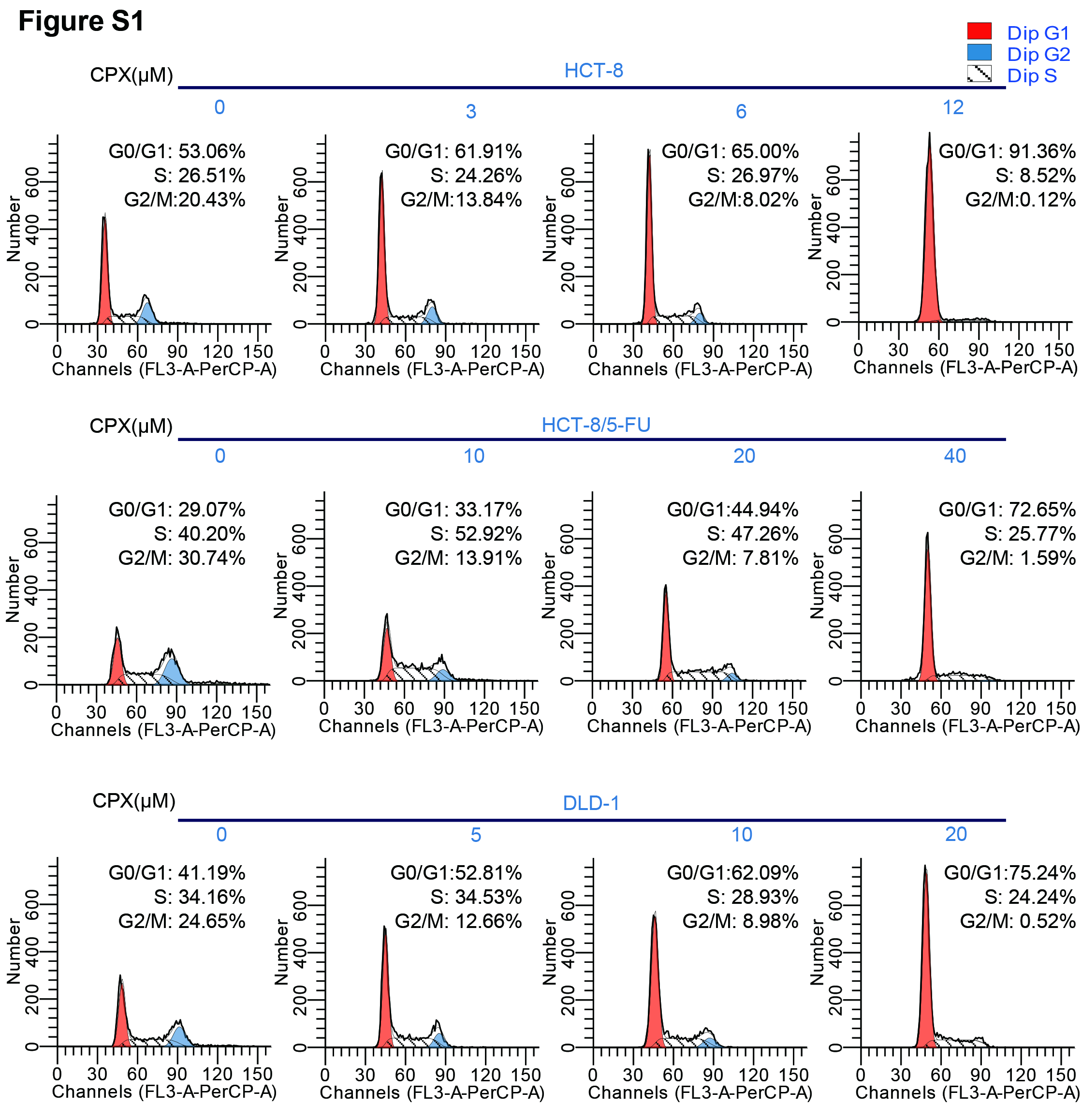

Supplement: Supplementary file 2 — Figure S1 [file 41419_2020_2779_MOESM2_ESM.tif]

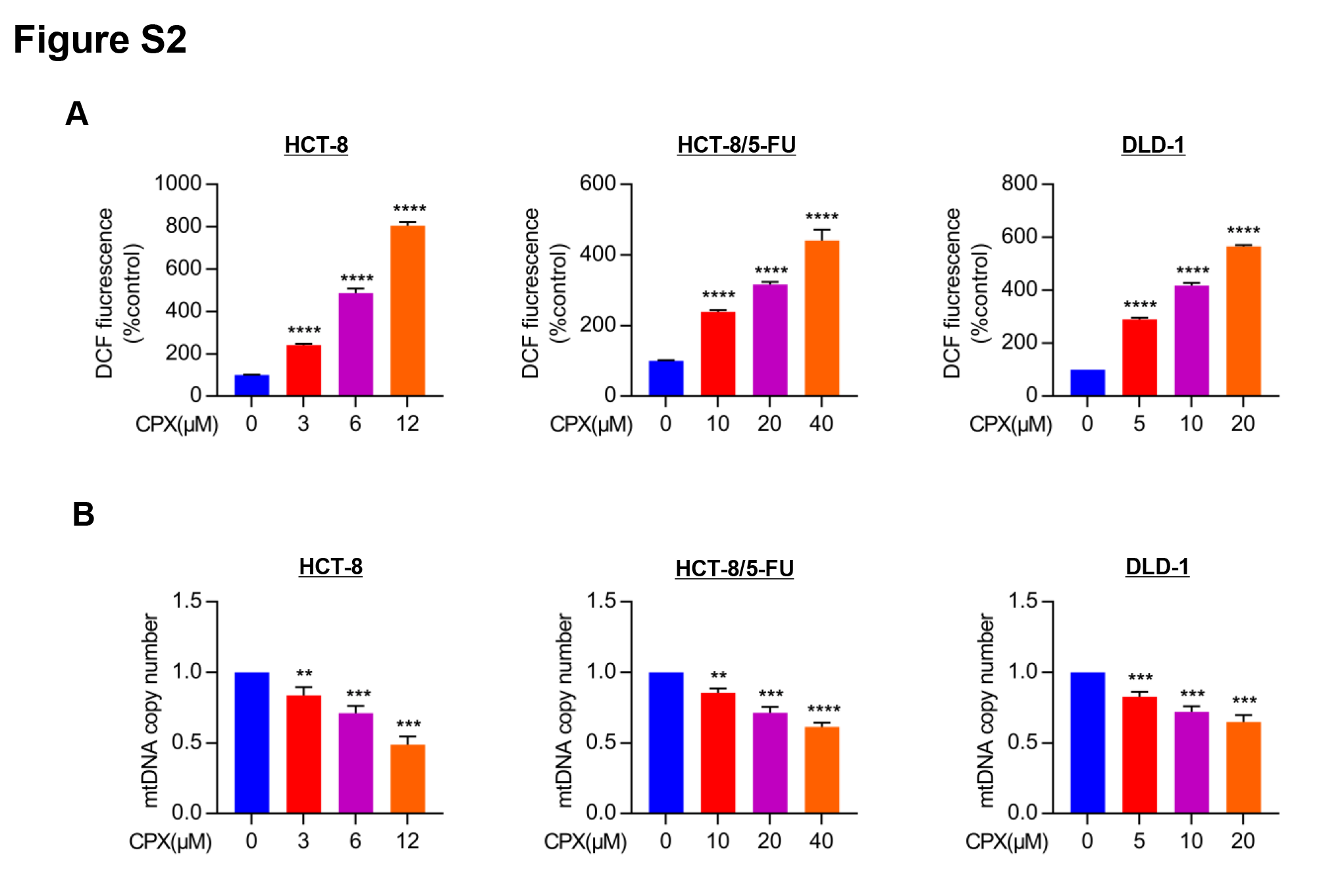

Supplement: Supplementary file 3 — Figure S2 [file 41419_2020_2779_MOESM3_ESM.tif]
