# Supplementary material for: Ciclopirox activates PERK-dependent endoplasmic reticulum stress to drive cell death in colorectal cancer
Source: Cell Death Dis. 2020 Jul 27;11(7):582. doi: 10.1038/s41419-020-02779-1 (PMC7385140; doi:10.1038/s41419-020-02779-1)
Supplement: Supplementary file 4 — Supplementary Table 1 [file 41419_2020_2779_MOESM4_ESM.docx]

**Supplementary Table 1. Details of the antibodies used for Western-blot analyses.**

| **Antibodies** | **Source** | **Cat #** |
| --- | --- | --- |
| Anti-ATF4 | Abcam | ab184909 |
| Anti- COX2 | Abcam | ab110258 |
| Anti-Ki67 | Abcam | ab16667 |
| Anti- PARP | Abcam | ab191217 |
| Anti- SDHA | Abcam | ab14715 |
| Anti- UQCRC2 | Abcam | ab14745 |
| Anti-MMP9 | ABconal Technology | A0289 |
| Anti-p-CDK4-T172 | ABconal Technology | AP0593 |
| Anti-p-CDK6-Y13 | ABconal Technology | AP0326 |
| Anti- Retinoblastoma (Rb) | ABconal Technology | A3618 |
| Anti-E-Cadherin | BD Biosciences | 610182 |
| Anti-CDK4 | Bio-RAD | VWA00520 |
| Anti-CDK6 | Bio-RAD | VWA00022 |
| Anti-p-Cyclin D1 (Thr286) | Cell signaling Technology | 3300 |
| Anti-p-Rb (Ser807/811) | Cell signaling Technology | 8516 |
| Anti-CHOP | Cell signaling Technology | 5554 |
| Anti-eIF2α | Cell signaling Technology | 5324 |
| Anti-p-eIF2α | Cell signaling Technology | 3597 |
| Anti-Ero1-Lα | Cell signaling Technology | 3264 |
| Anti-N-Cadherin | Cell signaling Technology | 13116 |
| Anti-PERK | Cell signaling Technology | 5683 |
| Anti-PDI | Cell signaling Technology | 3501 |
| Anti-PKM2 | Cell signaling Technology | 3198 |
| Anti-Snail | Cell signaling Technology | 3879 |
| Anti-cyclin A | Santa Cruz | sc-751 |
| Anti-cyclin B1 | Santa Cruz | sc-245 |
| Anti-cyclin D1 | Santa Cruz | sc-20044 |
| Anti-MMP2 | Santa Cruz | sc-13595 |
| Anti- PCNA | OriGene | TA800875 |
| Anti-ATP5A | Proteintech | 14676-1-AP |
| Anti-COX4 | Proteintech | 11242-1-AP |
| Anti-NDUFA9 | Proteintech | 20312-1-AP |
| Anti-PGK1 | Proteintech | 17811-1-AP |
| Anti-HK2 | Zen Bioscience | 220458 |
| Anti-PFKM | Zen Bioscience | 505477 |
| Anti- LDHA | Zen Bioscience | 383054 |
| Anti-β-Actin | Abmart | P30002 |
