# Supplementary material for: Ciclopirox activates PERK-dependent endoplasmic reticulum stress to drive cell death in colorectal cancer
Source: Cell Death Dis. 2020 Jul 27;11(7):582. doi: 10.1038/s41419-020-02779-1 (PMC7385140; doi:10.1038/s41419-020-02779-1)
Supplement: Supplementary file 5 — Supplementary information [file 41419_2020_2779_MOESM5_ESM.pdf]

Supplementary Table 2. Sequences of primers used in q PCR for mtDNA copy number analysis

Genes	Forward 5′-3′	Reverse 5′-3′
Cyt b	CCC CAC AAA CCC CAT TAC TAA ACC CA	TTT CAT CAT GCG GAG ATG TTG GAT GG
18S ribosomal DNA	TAGAGGGACAAGTGGCGTTC	CGCTGAGCCAGTCAGTGT
